# Effect of three different veneering techniques on the stress distribution and in vitro fatigue behavior of core-veneer all-ceramic fixed partial dentures

**DOI:** 10.34172/joddd.2021.032

**Published:** 2021-08-25

**Authors:** Alexandre Luiz Souto Borges, Anna Karina Figueiredo Costa, Amanda Maria de Oliveira Dal Piva, Alana Barbosa Alves Pinto, João Paulo Mendes Tribst

**Affiliations:** ^1^Department of Dental Materials and Prosthodontics, São Paulo State University, Institute of Science and Technology, Brazil

**Keywords:** Computer-aided design, Dental materials, Fatigue, Finite element analysis

## Abstract

**Background.** The present study aimed to evaluate the influence of the veneering technique on the tensile stress distribution and survival of full-ceramic fixed dental prostheses (FDPs).

**Methods.** A three-dimensional model of an FDP was modeled on a second premolar and a second molar with a pontic between them for finite element analysis (FEA). The groups were divided according to the veneering technique: conventional stratification, rapid layer, and CAD-on techniques. A mesh control test determined the number of elements and nodes. The materials’ properties were attributed to each solid component with isotropic, homogeneous, and linear elastic behavior. For the in vitro fatigue test (n=30), the FDPs were cemented on dentin analog abutments and submitted to 2×10^6^ mechanical cycles (100 N at 3 Hz).

**Results.** Maximum principal stress showed that the connector between the pontic and the second molar concentrated higher stresses, regardless of the techniques: Rapid layer (6 MPa) > CAD-on (5.5 MPa) > conventional stratification (4 MPa). The conventional stratification technique concentrated high stresses at the interface between the framework and veneering ceramic (2 MPa), followed by the rapid layer (1.8 MPa) and CAD-on (1.5 MPa) techniques. The crowns fabricated using the rapid layer and CAD-on techniques exhibited a 100% survival rate, while the conventional stratification group had 0% survival.

**Conclusion.** Even with similar stress distribution between the veneering techniques, the conventional stratification technique was more prone to failure under fatigue due to higher defects incorporated than CAD-on and rapid layer techniques.

## Introduction

All-ceramic restorations are increasingly indicated as an alternative to full-metal or metal-ceramic prostheses.^1,2^ All-ceramic restorations can provide excellent mimicry of the optical properties of natural teeth and present adequate biocompatibility.^3^ Polished or glazed dental ceramics accumulate less biofilm,^4^ are less prone to failure,^5^ and exhibit low thermal conductivity associated with high abrasion resistance and color stability.^6,7^

Despite these favorable properties, there is concern regarding the fracture resistance of all-ceramic restorations in situations where masticatory loads are higher, such as fixed partial dentures in the posterior region. These concerns are most relevant for all-ceramic restorations fabricated from dissimilar materials in the form of layered core-veneer structures. Systematic reviews of the clinical survival of contemporary all-ceramic core-veneer prostheses highlight that mechanical failures occur primarily in the form of chipping or delamination in the veneering layer.^1,8^ Based on a longitudinal 5-year evaluation, this treatment modality is similar (93.8%) to metal-ceramic crowns (95.7%).^1^ However, it is far from the ideal situation, and consequently, different approaches have been developed to prevent mechanical failures.

Layered core-veneer all-ceramic restorations are fabricated from a high-strength polycrystalline core that is partially or fully covered with an amorphous glass-ceramic layer applied to enhance restoration esthetics. The veneer layer has been traditionally applied manually by shaping and condensation of a slurry formed by the ceramic powder and modeling liquid.^9^ Compared with chairside dentistry, manual veneering is time-consuming and susceptible to operator-induced variability.^10^ Defects can be introduced in the form of voids, porosity, and micro-cracks within the veneer layer or at the interface with the core.^9,11^ In addition, sintering of the veneer ceramic introduces residual stresses that arise due to mismatches in the thermal compatibility of the core and veneer ceramic.^12^ The presence of defects and residual stress induces sub-critical crack propagation, making the restoration susceptible to delamination and fracture.^11,13^

Heat pressing techniques using highly controlled temperature and pressure conditions have been employed to apply the veneering ceramic to a prefabricated core to mitigate some of the limitations associated with manual veneering. This approach can reduce the incidence of internal defects in the veneer layer and allows ceramic materials with improved mechanical properties to be used for veneering, including leucite glass-ceramics^14,15^ or lithium disilicate glass-ceramics.^13,16^ However, heat pressing also results in residual stress states and requires additional manual steps, including wax pattern generation.

As an alternative, computer-aided design/computer-aided manufacture (CAD/CAM) workflow has been developed to fabricate both the veneer and framework structures from homogenous ceramic blocks that have been manufactured in optimized conditions.^17^ The two CAD/CAM parts are subsequently joined using a composite resin layer (Rapid Layer Technique, VITA Zahnfabrik, VITA Zahnfabrik, Bad Säckingen, Germany)^18^ or with a low fusing temperature glass-ceramic (IPS e.max CAD Veneering Solutions, CAD-on technique, Ivoclar Vivadent, Schaan, Liechtenstein).^19^ The fracture resistance of the resulting ceramic bilayer will depend on many geometric, material, and processing variables,^18,19^ and the interface between the two layers for core-veneer all-ceramic restorations has been identified to be important in determining the fracture behavior.^18,20^ It is proposed that effective stress transfer mediated by a stable joint between layers promotes greater fracture resistance under masticatory forces.^21-28^ However, these techniques have not been compared before regarding stress distribution and fatigue survival.

This study aimed to evaluate the influence of three different veneering techniques on the tensile stress distribution and survival of full-ceramic fixed dental prostheses (FDPs). The first null hypothesis was that the in vitro FDP survival is independent of the technique used to create the core-veneer interface. The second null hypothesis was that there is no difference in the stress distribution between the models.

## Methods

### Finite element analysis – 3D model generation

**Figure 1:**
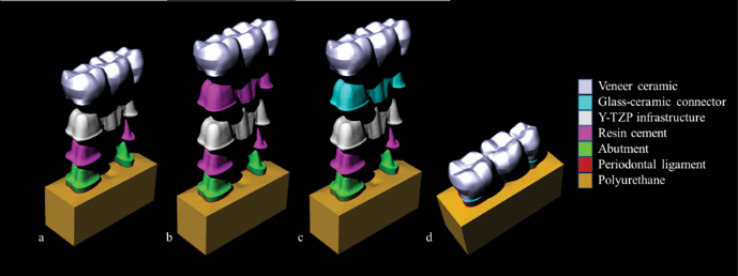
Schematic illustration of the modeling procedures according to the manufacturing technique to join the veneer and Y-TZP core: (a) conventional layer stratification technique. (b) Rapid layer technique. (c) CAD-on technique. (d) All the groups corresponded to a geometrically identical three-unit FDP.

A three-dimensional (3D) model of an FDP was generated following the ‘Biocad’ protocol.^21^ A 3D model of the mandibular second premolar and a second molar abutments was selected from the UNESP São José dos Campos database. A pontic replacing the mandibular first molar was modeled using relevant anatomic data. The resultant STL (stereolithography) files were transferred to modeling software (Rhinoceros 4.0, McNeel North America, Seattle, USA). Polylines were drawn on the STL image, following the main anatomical landmarks which referenced the surface. The delimited surfaces were subsequently joined, forming closed solids and generating volume to the structures. The generated 3D model was geometrically representative of the model subsequently used for the *in vitro* fatigue test. The prepared abutment teeth exhibited 6° of convergence between axial walls with the finishing line as a rounded shoulder with a radius of curvature of 0.5 mm. Groups were distributed according to the veneering technique for the yttria-stabilized tetragonal zirconia polycrystal (Y-TZP) core. Figure 1 is the schematic representation of conventional stratification, rapid layer, and CAD-on techniques. For all the groups, a resin cement thickness of 100 µm was introduced between substrate and FDP.^29^ For the rapid layer technique, an identical resin cement layer was simulated between the Y-TZP core and veneer. For the CAD-on technique, the glass-ceramic connector was modeled with 100 µm thickness.

The geometries were imported into analysis software (ANSYS 17.2, ANSYS Inc., Houston, USA) in STEP (Standard for the Exchange of Product Model Data) format, and a mesh was generated using tetrahedral elements. A mesh convergence test was performed to determine the appropriate mesh density (number of elements and nodes) with a threshold level set at 10% to reduce discretization error. The material properties attributed to each solid component reflected isotropic, homogeneous, linear, and elastic behavior. The elastic modulus and Poisson ratio assigned for each material were based on the literature (for fixed variables in the model) or were experimentally determined (Table 1).^26-28^ Elastic constants for the ceramics and cements were determined from the non-destructive characterization of vibration frequencies using pulsed excitation. For that, five rectangular specimens (20×10×4 mm) of each ceramic were prepared according to the manufacturer’s instructions and polished sequentially with P600-, P800-, and P1200-grit SiC abrasive to a consistent surface finish.^22^ During the test, the supported rectangular specimen was excited manually using a light impact hammer, and the acoustic response was recorded to determine the natural vibration frequencies (ATCP Sonelastic, Physical Engineering, Ribeirão Preto, SP, Brazil). Measurements were repeated 10 times for each sample, and the mean elastic modulus and Poisson ratio were calculated.^22^ Following material property assignments and mesh sensitivity analysis, all contacts were considered ideally bonded, and the fixation occurred in the base of the substrate.^23^ A 100-N load was applied on the pontic in contact with the grinding slope of the mesio-vestibular cusp of the pontic.

**Table 1:** Elastic constants of the materials used in this study

**Materials**	**Elastic modulus (GPa)**	**Poisson ratio**	**Reference**
Feldspathic ceramic (VM9, VITA Zahnfabrik)	65.0 ± 6.0	0.30	Experimentally determined
Leucite ceramic (Vitablocs TriLuxe forte, VITA, Zahnfabrik)	70.7 ± 5.0	0.31	
Lithium disilicate (IPS e.max CAD, Ivoclar Vivadent)	95 ± 3.4	0.30	
Y-TZP	200	0.31	27
Resin cement (Panavia F, Kuraray)	9.2 ± 2.0	0.28	Experimentally determined
Glass-ceramic connector (Crystal Connect, Ivoclar Vivadent)	70 ± 6.2	0.31	
Dentin analogue (G10)	18	0.30	28
Periodontal Ligament	0.068	0.45	29
Polyurethane resin (Axson F16, Axson)	3.6 ± 0.4	0.30	Experimentally determined

### In vitro fatigue simulation

#### Sample preparation

**Figure 2:**
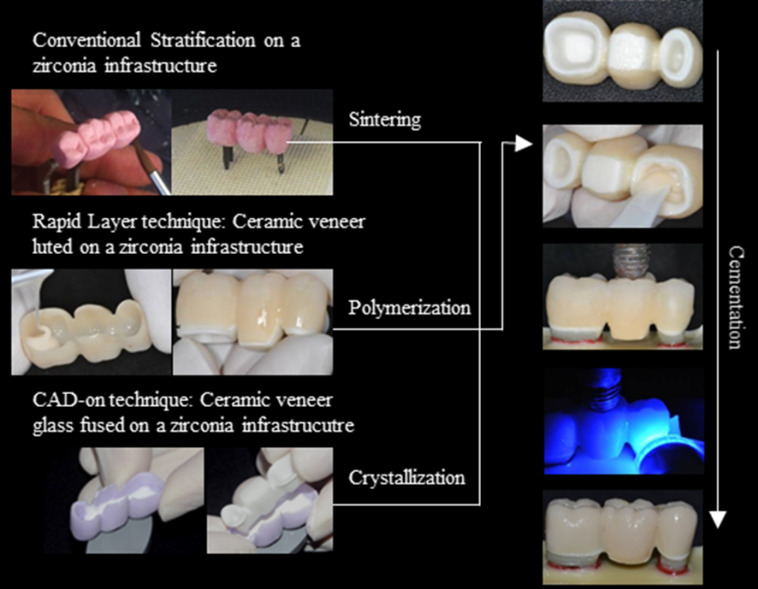
Study design. Three different manufacturing techniques were used to create veneered Y-TZP bilayered FDPs: Conventional ceramic layering (stratification), Vita Rapid Layer, and IPS e.max CAD-on techniques. Prosthesis geometry was nominally identical regardless of the technique used. Following fabrication, prostheses were adhesively cemented on the prepared FDP abutment specimens.

For in vitro fatigue simulation, a three-unit FDP model was generated using the same geometry and materials used for finite element analysis (FEA). The abutment teeth were machined from G10 Epoxyglas (Accurate Plastics, Massachusetts, USA), previously advocated as a dentin-analog material.^24^ Following machining, the root portion of each abutment tooth was coated with a thin wax layer before being accurately located into a silicone matrix which was then filled with a chemically activated polyurethane resin (Axson F16, Axson, São Paulo, Brazil; Batch number: 0010018040), covering the entire tooth root. Polyether impression material (Impregum F, 3M-ESPE, Minnesota, USA; batch number: 421709) was inserted into the simulated ‘alveolus’ as an analog material for the periodontal ligament.^25^ Excess impression material was removed with a #11 scalpel blade. Thirty FDP abutment samples were obtained and randomly assigned to three groups. The abutments were scanned, and identical core frameworks were designed in CEREC 3.8 software (Dentsply Sirona, USA). Y-TZP cores were machined, and the prostheses were fabricated using three different approaches outlined in Figure 2.

### Conventional stratification technique

For the stratification technique, 10 Y-TZP (Vita InCeram YZ, (VITA Zahnfabrik, Bad Säckingen, DE; Batch number: 23611) were selected. No further surface treatments were applied before manual veneering with feldspathic ceramic (Vita VM9, VITA Zahnfabrik, Bad Säckingen, DE; Batch number: 16820). The veneering ceramic was manually applied and shaped by a single operator skilled in the stratification technique. Mock-ups were used to ensure that the shape generated followed the 3D model anatomy. Following the application, the ceramic was sintered in Vacumat 6000 MP furnace (Vita Zahnfabrik, Bad Säckingen, DE) according to the manufacturer’s recommended firing cycle (500^o^/6 min, t ↑ 55º/min up to 910º/min).

### Rapid layer technique

For the rapid layer technique, ten Y-TZP frameworks were selected and particle-air-abraded with 50-μm Al_2_O_3_ particles under a pressure of 2.5 bar at a distance of 10 mm for 10 seconds. The veneer layer was machined from a leucite glass-ceramic (Vitablocs TriLuxe forte TF-40/19, VITA Zahnfabrik, Bad Säckingen, DE; Batch number: 32330) according to the manufacturer’s instructions for the rapid layer approach. The inner surface of the veneer layer was acid-etched with 10% HF acid (Condac Porcelana, FGM, Joinville, BR; Batch number: 290414) for 1 minute, washed thoroughly for 30 seconds, and then air-dried. A silane priming agent (3-MPS, Monobond Plus, Ivoclar Vivadent, Schaan, Liechtenstein) was then applied for 1 minute. The veneer layer was adhesively cemented to the air-abraded Y-TZP core with the dual-cured resin cement, Panavia F (Kuraray, Tokyo, JPN; Batch number: 051234). The two layers were pressed together under light manual pressure to remove excess cement, followed by additional photo-activation for 40 seconds on each face (Poly wireless – KAVO, 1100 mW/cm^2^ - Brazil Ind. Com. Ltda, Joinville, BR).

### CAD-on technique

Y-TZP FDP frameworks were fabricated from IPS e.max ZirCAD (Ivoclar Vivadent, Schaan, Liechtenstein; Batch number: P55259) using the same CAD geometry for the other groups. The veneer layer was machined from IPS e.max CAD (Ivoclar Vivadent, Schaan, Liechtenstein; Batch number: R64956), which is a partially sintered lithium silicate glass-ceramic. A ‘glass-ceramic connector’ material, IPS e.max CAD Crystall./Connect (Ivoclar Vivadent, Schaan, Liechtenstein; Batch number: R59519), was used to join the core and veneer layers. The glass-ceramic connector was applied to the intaglio surface of the veneering ceramic using a vibration table (Ivomix, Ivoclar Vivadent, Schaan, Liechtenstein) to eliminate bubbles. Both layers were then pressed together under manual pressure to remove excess material. The samples were subjected to a crystallization firing cycle (403º/6 min, t ↑ 60º/min - T 850º/10 min - Vac1 770º - Vac2 850^o^) in a Programat EP 3000 furnace (Ivoclar Vivadent Inc. Amherst, New York, USA).

### FDP cementation and in vitro cyclic fatigue testing

Before the luting procedure, the FDP outer surfaces were polished, following the manufacturer’s instructions. The dentin analog abutment teeth were etched with 10% HF acid, washed, and dried. The FDPs were then cemented using a dual-activated resin cement (Panavia F, Kuraray, Toquio, Japan, Batch number: 051234) under the constant pressure of 750 g for 10 minutes with the aid of an adapted parallelometer (BioArt, São Carlos, SP, Brazil).^25^ Excess cement was removed before light-curing at the restoration margins for 40 seconds on all the faces of each abutment (Poly wireless - KAVO 1100 mW/cm^2^ - Brazil Ind. Com. Ltd. Joinville, BR). After 10 minutes, the samples were stored in distilled water at 37±1°C for five days.

Cyclic fatigue testing was performed in a masticatory cycle simulator (Biocycle V1 Mechanical Cycler, Biopdi, São Paulo, BR). The test samples were positioned on a rigid metal base in water (37±1°C) to form a 90° angle between the horizontal plane and the loading tip. A 6-mm-diameter stainless steel indenter was positioned in contact with the grinding slope of the mesio-vestibular cusp of the pontic. A thin layer of ‘rubber dam’ was placed between the indenter and the occlusal surface to reduce contact stresses. Each sample was subjected to an axial load cycle at 3 Hz, with a peak load of 100 N and a maximum load duration of 1.7 seconds. After each period of 500 000 cycles, the FDP surfaces were stained with a liquid penetrant designed to non-destructively identify crack propagation (Metal-Chek, Bragança Paulista, São Paulo, BR) and inspected using a stereomicroscope (Stereo Discovery V12, Carl Zeiss AG, Oberkochen, DE). Failure was defined and classified as: visible cracks in the veneering ceramic, chipping of the veneering ceramic, delamination exposing the core, radial cracks reaching the core, and catastrophic fracture. Testing was performed up to two million cycles, and the specimens that did not fail were considered suspended.

### Stereography and scanning electron microscopy

Representative specimens from each group were randomly selected after fatigue testing and inspected using a polarized light stereomicroscope (Stereo Discovery.V20, Carl Zeiss, LLC, USA) and a scanning electron microscope (SEM; Inspect S50, FEI, Czech Republic). Representative specimens were sputter-coated with gold for 180 seconds at 40 mA, creating a 30-nm-thick coating layer and then examined under different standard SEM magnifications operated at 20 kV using secondary electron detection.

### Statistical analysis

Distributions of maximum principal stress from FEA were visualized in the form of color plots. For fatigue testing, the loading step associated with specimen failure was recorded and used for survival analysis. After tabulation of the data in a survival table, the Kaplan-Meier test followed by Wilcoxon and Log Rank tests were performed in Minitab 17 statistical software (Pennsylvania, United States of America) at α = 0.05.

## Results

### Finite element analysis

**Figure 3:**
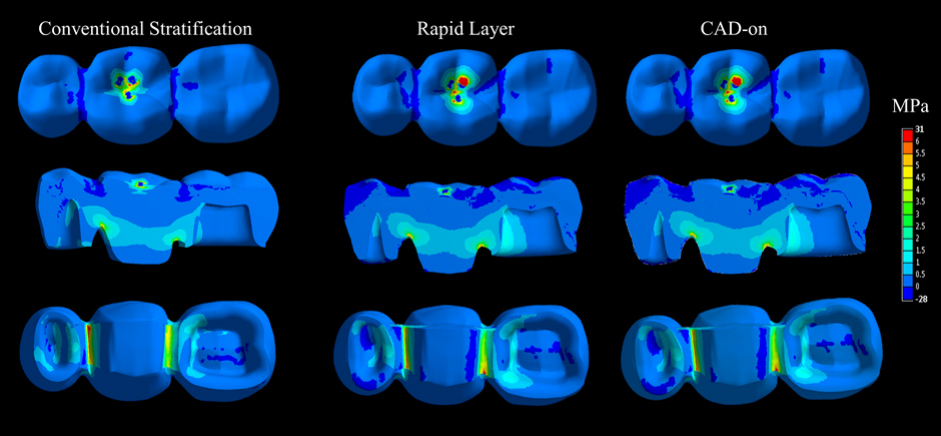
Distribution of maximum principal stresses (MPa) with the prosthesis for the three FDP veneering techniques.

The simulated maximum principal stress (MPa) for the three core-veneer FDP are shown in Figure 3. Stress concentration occurred at the site of load application within the veneering ceramic and at the connector region between the pontic and abutment teeth. For an applied load of 100 N, the simulated maximum principal stresses for all the three FDP were low (< 6 MPa), and a similar pattern of stress distribution was generally observed. Regardless of the veneering technique, the highest stress peak occurred in the bottom surface of the connector region and was located with the automated maximum label after the processing analysis. Modifying the interface between the veneer and core resulted in minor differences in stress at connector regions. The most notable asymmetry in stress concentration between mesial and distal connector regions was observed for the conventional stratification technique. Further minor changes in the stress distribution pattern were observed at the interface between the core and veneering ceramic.

### In vitro cyclic fatigue testing

**Figure 4:**
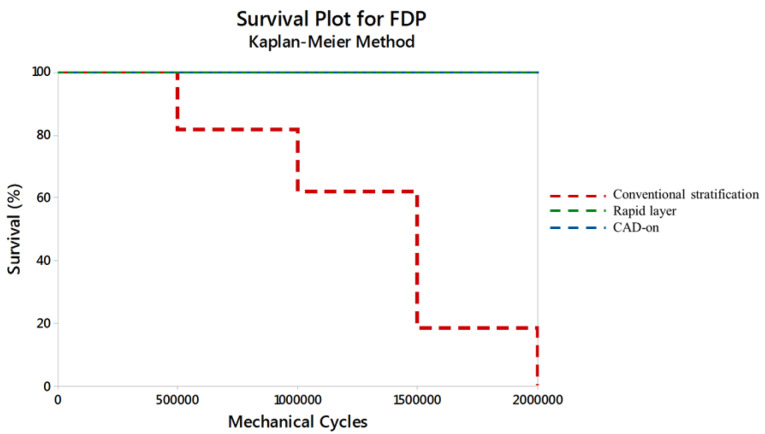
Survival plot versus the number of mechanical cycles.

A significant difference was observed between the fatigue behavior of all FDPs, as shown in the survival plot (Figure 4). Wilcoxon (χ^2^ = 189.4) and log-rank (χ^2^ = 205.9) tests identified a significant difference between cycles to failure for the FDPs (*P* < 0.01). All the FDPs fabricated using the rapid layer and CAD-on techniques survived 2×10^6^ cycles, while all the FDPs fabricated by conventional stratification failed.

**Figure 5:**
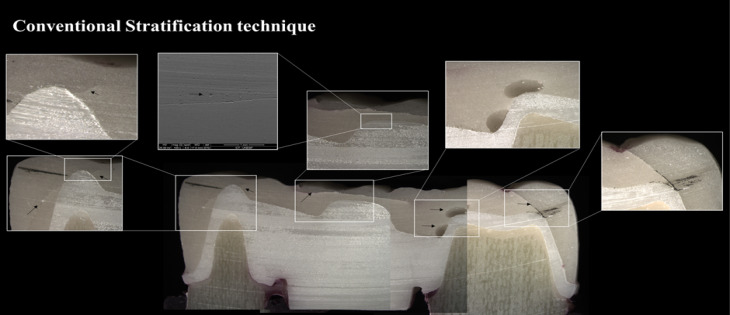
A representative sectioned specimen from the conventional stratification technique showing visible cracks (black arrows) in the veneering ceramic after 500,000 cycles. Inspected under a polarized light stereomicroscope (magnification of ×25) and SEM (magnification of ×100).

**Figure 6:**
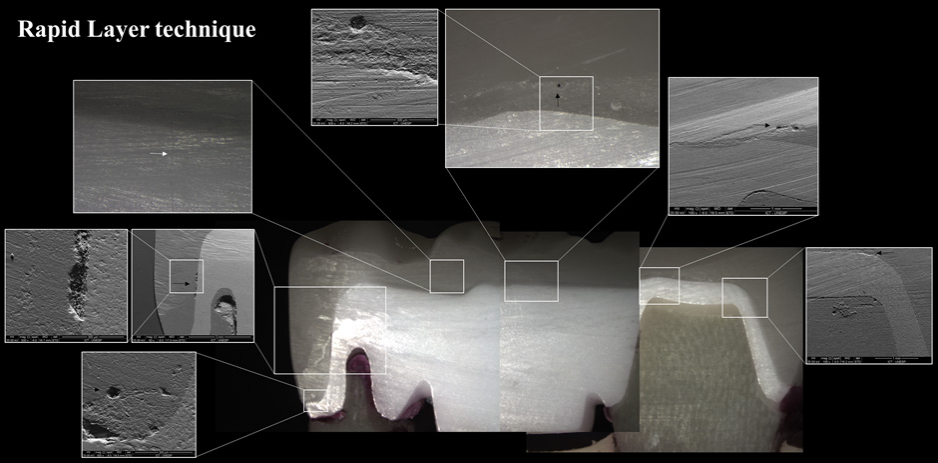
A representative sectioned specimen from the rapid layer technique inspected under a polarized light stereomicroscope (magnification of ×25) and SEM (magnification of ×50, ×100, and ×500), showing cracks within the veneering ceramic (white arrow) and bubbles (black arrows) in the cement layer between the zirconia core and veneering ceramic.

**Figure 7:**
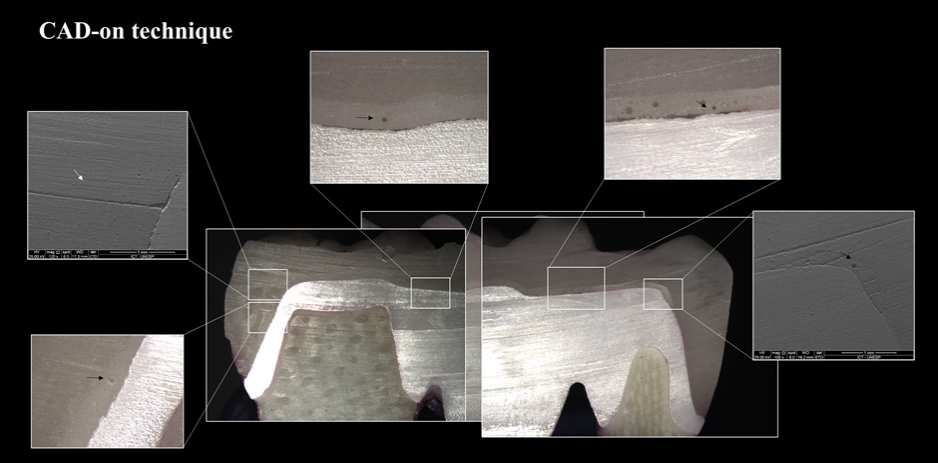
A representative sectioned specimen from the CAD-on technique was inspected under a polarized light stereomicroscope (magnification of ×25) and SEM (magnification of ×100 and ×120) showed the prevalence of bubbles (black arrows) in the glass between the zirconia core and veneering ceramic.

The test specimens fabricated using a conventional stratification technique showed visible cracks in the veneering ceramic after 500 000 cycles. In contrast, the test specimens fabricated by the rapid layer and CAD-on techniques exhibited no visible defects even after 2×10^6^ cycles. Following testing, the specimens were cross-sectioned (Figures 5-7) to allow the visualization of the interface. In particular, voids were observed in the stratification technique (Figure 5). In samples fabricated by the rapid layer technique, delamination of the veneering ceramic between the resin cement and the Y-TZP infrastructure was observed (Figure 6). The glassy connector material used in the CAD-on technique remained adhered to both Y-TZP and veneer; following fatigue, only minor defects (porosity) were observed without visible cracks (Figure 7).

## Discussion

This in vitro study aimed to compare survival following cyclic loading of geometrically and nominally identical FDPs fabricated with different strategies to join Y-TZP core and veneering ceramic. The first null hypothesis was rejected since significantly different survival rates were identified between conventional layering and the CAD-on/rapid layer techniques. The second null hypothesis was accepted since no difference was observed in the stress maps for all the veneering techniques.

Clinical studies have shown an acceptable success rate for veneered Y-TZP restorations; however, the most common cause for early failures is veneer material chipping or delamination.^8,30^ This study demonstrated that the technique used to create the interface between veneering ceramic and Y-TZP core could affect FDP survival. FDP fabricated using the conventional stratification technique exhibited cracks and voids near the core-veneer interface and within the veneering ceramic, and all the test specimens failed before 2×10^6^ cycles. In contrast, all the specimens fabricated with a CAD/CAM veneer layer survived until 2×10^6^ cycles (Figure 4). Mechanical cycling, when performed on test specimens immersed in a wet environment, consists of a more representative condition that leads to slow crack growth, decreasing the ceramic fracture resistance over time.^31,32^ Many cyclic fatigue studies are short due to the time constraints associated with this method. In this study, approximately 8.5 years of clinical use was simulated, exceeding the five years (~1.2 million cycles) that is commonly reported.^33,34^

Previous studies have reported that the microtensile bond strength between veneering ceramic to a Y-TZP core using the CAD-on technique is superior (44 MPa) to that achieved using a heat/press technique (14 MPa).^35^ The authors also suggested that the failure of the CAD‐on technique is less likely to occur within the veering ceramic itself, which was corroborated by the findings of this study. It was reported that the vibration of the connecting glass between the core and the veneer could incorporate bubbles and create voids along the interface.^35^ In this study, voids were observed according to the microscopic analyses; however, they did not affect the prosthesis survival until the test suspension. In addition, according to the manufacturer, a vibration system (Ivomix) is indicated to remove the air within this glass layer, reducing the defects in this layer.^36^ Thus, in addition to the residual stress, other factors might be essential for the beginning of the fracture in the stratified group.

Most of the FEA studies in dentistry are limited due to the absence of defect simulation using homogeneous material. However, this is also a limitation of the present study. The stress distribution results were compared with the *in vitro* results in this study, indicating that some simplifications made in the computational model did not allow predicting the lower survival of the conventional group since the colorimetric maps showed a very similar stress distribution between the groups. As observed in microscopic analyses, there were voids and cracks inside the veneering material. Therefore, as the FEA limitation, it did not allow total fidelity in the failures of stratified ceramics. It is important to consider that the maximum tensile stress will always occur in the region close to the load application due to the Saint-Venant effect. Thus, the volume of the material affected by this stress concentration depends on the material’s elastic modulus.^37,38^ The presence of a connector in the FDP suggests that the stress in this area can be more concentrated than the interface layer, explaining the differences between previous simulations^17^ and the present study. Probably, without the voids, the three groups would have survived similarly.

Further studies should be developed to investigate the FDP behavior under simulation of sliding forces, thermal or pH variations,^39^ and different luting agents^40^ since these factors have already been reported to modify the biomechanical response of dental materials. In addition, the numerical model presented limitations as the isotropic behavior in the mechanical properties and the homogeneous volume, which did not reproduce defect distribution incorporated during the FDP manufacturing.^23^ Future fatigue life studies and clinical evaluations might complement the results or evaluate the FDP’s longevity according to the described techniques.

## Conclusion

Regardless of the similarity observed, using FEA for the stress distribution between the groups, the conventional stratification technique for FDP manufacturing showed higher susceptibility to failure under fatigue due to the higher number of defects incorporated during manufacturing compared to the CAD-on and rapid layer techniques. Therefore, both CAD-on and rapid layer techniques showed promising behavior as FDP manufacturing techniques.

## Authors’ Contributions

The concept and the design of the study were developed by ALSB, AKFC, AMODP, and JPMT. The *in vitro* method was carried out by AKFC and ABAP. The *in silico* method was carried out by ALSB, AKFC, and JPMT. Data entry and statistical analyses were carried out by ALSB, AMODP, and JPMT. The manuscript was written by ALSB, AMODP, ABAP, and JPMT. All the authors participated in the literature review. All authors have read and approved the final manuscript

## Acknowledgments

The authors would like to thank São Paulo Research Foundation (FAPESP) for the grants numbers 12/11095-0 and 14/00668-4.

## Funding

This research was funded by São Paulo Research Foundation (FAPESP) under the grant numbers 12/11095-0 and 14/00668-4.

## Competing Interests

The authors declare no competing interests with regards to the authorship and/or publication of this article.

## Ethics Approval

Not applicable.
